# Books Reviewed

**Published:** 1863-02

**Authors:** 


### BOOKS REVIEWED.

*Consumption in New England, or Locality one of its Causes. An Address delivered before the Massachusetts Medical Society. By* HENRY I. BOWDITCH, M. D. *Boston:* TICKNOR & FIELDS, 1862.

The two following propositions contain the essential points of this Address:

"*First.*—A residence on or near a damp soil, whether that dampness be inherent in the soil itself, or caused by percolation from adjacent ponds, rivers, meadows, marshes or springy soils, is one of the primal causes of consumption in Massachusetts, probably in New England, and possibly in other portions of the globe.

*Second.*—Consumption can be checked in its career, and possibly, nay, probably, prevented in some instances, by attention to this law."

"I lay down now before you, as among my *medical axioms*, the following statements:

1st—*Consumption is not, as some writers have contended, epidemic equally in every part of New England;* but there are some localities where it is very rife, and others where it is vastly less destructive than in the State at large.

2d—*There is a law*, hitherto scarcely noticed, or but vaguely hinted at by one or two individual writers, but (as I believe) *never proved* until now, *which is one of the main causes, if not the sole cause, of this unequal topographical distribution of consumption in New England.*

3d—*This law is intimately connected with, and apparently dependent on, the humidity of the soils, on or near which stand the towns, villages, or even single houses, where consumption prevails.*

4th—*The existence of this law of soil-moisture, as one of prime causes of consumption in New England*, can be be proved, as I think, by several lines of argument, resting on actual facts obtained either from public or private records, statistical data, or the opinions of physicians, practicing medicine in various parts of New England."

The proofs of the above propositions, and the arguments which sustain them, constitute the Address. A map of Massachusetts is made, showing the locations where consumption is rife generally in the town, or in localities or houses, more so, than in the town generally; also where the disease is quite common in every part of the town, and still more so in certain localities, and also where it is more rarely seen in the town as a whole, but in which are found certain localtties peculiarly affected by it; and lastly towns in which consumption is rare. The Address and accompanying explanations and illustrations, make up a book of over one hundred pages, which shows great expenditure of time and thought. The reputation of the author as a physician practically and extensively engaged in the special study and treatment of diseases of the lungs, will insure consideration for the doctrines he advocates; while the ability with which he has presented his facts and arguments, will prove almost irresistible in gaining assent to the propositions he has attempted to prove.

---

*The Retrospect of Practical Medicine and Surgery; being a half-yearly Journal, containing a retrospective view of every discovery and practical improvement in the medical sciences. Edited by* W. BRAITHWAITE, M. D., *late Lecturer on Midwifery, and the Diseases of Women and Children, at the Leeds School of Medicine, etc, and* JAMES BRAITHWAITE, M. D., *London. New York: Published by* W. A. TOWNSEND.

Every practicing physician should subscribe for this book. We have no occasion to speak of its practical value, since all physicians are fully aware that every valuable improvement in practice of medicine and surgery will be more or less fully considered in this half yearly abstract. It is our opinion that there is no one in the practice of our art who can afford to neglect subscribing for Braithwaite's Retrospect.

---

